# Antimicrobial inhibitory activity of aqueous, hydroalcoholic and alcoholic extracts of leaves and stem of Daphne mucronata on growth of oral bacteria

**DOI:** 10.3205/dgkh000301

**Published:** 2017-10-02

**Authors:** Kolsoom Shirzadi Karamolah, Fatemeh Mousavi, Hassan Mahmoudi

**Affiliations:** 1 Islamic Azad University, Boroujerd Branch, Lorstan, Iran; 2 Microbiology Department, Hamadan University of Medical Sciences, Hamadān, Iran

**Keywords:** Daphne mucronata, antimicrobial inhibition, agar disk diffusion method, minimum inhibitory concentration

## Abstract

**Background:** Plants are a source of potential anti-infective agents. *Daphne mucronata* is a shrub in the family Thymelaeaceae, which has therapeutic effects. The aim of the present study was to evaluate the antimicrobial activity of aqueous, hydroalcoholic and alcoholic extracts of the leaves and stem of *Daphne mucronata* on the growth of oral bacteria.

**Materials and methods:** Leaves and stem of *Daphne mucronata* were collected from the Zagros Mountains, Lorestan, Iran. They were air dried in the shade. Aqueous, hydroalcoholic and alcoholic extracts of *Daphne mucronata* were made by using classic techniques for solvent extraction of plant material. The antimicrobial effects of the* Daphne mucronata* extracts were evaluated using the agar disk diffusion method (ADDM) and the minimum inhibitory concentration (MIC). The data were analyzed using Duncan's test and ANOVA.

**Results: **The results showed that the antimicrobial activity depended on the type of extract. The alcoholic extract of *Daphne mucronata* had the highest antibacterial activity and the highest effect on *Streptococcus mutans*. The aqueous extract of the plant had no effect on bacterial growth.

**Conclusion:** On the basis of the current results, the alcoholic extract of *Daphne mucronata* might be promising as a natural antimicrobial agent and as a medicine for the prevention and control of the growth of *Streptococcus mutans*.

## Zusammenfassung

**Zielsetzung:** Pflanzen sind Quelle potentiell antimikrobiell wirksamer Verbindungen. Zielsetzung der vorliegenden Studie war die Bestimmung der antimikrobiellen Hemmkonzentration wässriger, hydroalkoholischer und alkoholischer Extrakte aus Blättern und Stamm von *Daphne mucronata* gegenüber oralen Bakterien.

**Material und Methode:** Blätter und Stamm von *Daphne mucronata* wurden im Zagrosgebirge in der Provinz Lorestan, Iran, gesammelt. Sie wurden im Schatten getrocknet. Die Extrakte wurden mittels klassischer Technik zur Lösungsmittelextraktion aus Pflanzen gewonnen. Die antimikrobielle Wirkung wurde im Agarplättchendiffusionstest und als minimale Hemmkonzentration ermittelt. Die Ergebnisse wurden mittels Duncan's und ANOVA Test analysiert.

**Ergebnisse:** Die antimikrobielle Wirksamkeit war abhängig von der Art der Extraktion. Der alkoholische Extrakt von Daphne mucronata war am wirksamsten mit der höchsten Effektivität gegenüber Streptococcus mutans, während der wässrige Extrakt unwirksam war.

**Schlussfolgerung: **Aufgrund der Ergebnisse könnte der alkoholische Extrakt von *Daphne mucronata* ein geeigneter Kandidat im Rahmen der Erforschung natürlicher antimikrobieller Wirkstoffe und speziell zur Prävention von *Streptococcus mutans* aussichtsreich sein.

## Introduction

Bacterial resistance to antibiotics has become a global problem. In recent years, much attention has been focused on the use of herbal medicine, due to fewer side effects [1]. The problem of antibiotic resistance is based on many factors, e.g., inappropriate use of antibiotics [2]. Owing to the beneficial properties of medicinal plants in the treatment of diseases and with regard to the availability and compatibility with the human immune system, their use is on the rise [3], [4], [5]. *Daphne mucronata* is a wild shrub of the family Thymelaeaceae, which is found in many parts of Iran [6]. This plant has been traditionally used to treat skin diseases and cancer. The daphnetin 8 glycoside obtained from *D. mucronata* might have cardiotoxic effects [7], [8], [9]. Cytotoxic activity of a hydroalcoholic extract of *D. mucronata* on different cells has been reported previously, mostly on lung cancer cells [10], [11], [12], [13]. *D. mucronata* is used in the northern areas of Pakistan for patients suffering from hot flashes, arthritis, fever, and muscle pain, as well as for topical treatment of inflammation and to treat gastric ulcers, rheumatism, dental decay and toothache [14], [15]. Studies have shown that *Daphne spp.* are a potential source of anti-biofilm agents [16], [17]. *Daphne gnidium* grows in Mediterranean regions, and extracts of its leaves and bark have been shown to possess antimicrobial and germicidal activity [11]. Therefore, this study aimed to investigate the therapeutic effects of *Daphne mucronata* as an alternative to synthetic drugs and chemical mouthwashes.

## Methods

### Collection of plant materials

The leaves and stems of* Daphne mucronata* were collected in the spring and summer (April–July 2015) from the Zagros Mountains, Lorestan, Iran and were authenticated by Department of Pharmacognosy and Pharmaceutical Biotechnology, School of Pharmacy, Hamadan University of Medical Science, Hamadan, Iran. 

### Extraction

Aqueous, hydroalcoholic and alcoholic extracts of the plant were prepared by soaking the dried leaves and stems using the percolation method. 

For the aqueous extract, 10 g of leaf powder were soaked in 100 ml of boiled, distilled water for 2 h at 60–70°C. The decoction was filtered and evaporated in a water bath at 50–60°C to yield a solid extract.

The ethanol extract was prepared using the same protocol. Dried, powdered plant materials were subjected to extraction using 95% ethanol and hydroalcohol (50% ethanol) for 6 h at 37°C. The extraction procedure was repeated thrice. The combined ethanolic and hydroalcoholic extracts were pooled and evaporated dry under reduced pressure at 40°C with rotator vacuum evaporator. Therefore, residual alcohol cannot affect the results. The percentage yields of the crude extracts were found to be 14.98% w/w for 95% ethanolic and 15.58% w/w for hydroalcoholic extracts. 

Concentrations of 6.25, 12.5, 25, 50, and 100 mg/ml of the leaf and stem extract solution were dissolved in dimethyl sulfoxide (DMSO), and 30 µl of each concentration was inoculated on to a blank disk [18]. Next the disks were kept at 25 °C for 5 hours for drying.

### Test organisms

In order to evaluate the antimicrobial properties, the gram-positive bacteria *Staphylococcus epidermidis *(ATCC12228), *S. aureus* (ATCC 10690) and *Streptococcus mutans* (ATCC 10672) and the gram negative bacteria *Neisseria sicca* (ATCC10721) and Pseudomonas aeruginosa (ATCC10205) were employed. The bacteria were cultivated on Mueller Hinton Agar (Merck, Germany) at 37°C for 24 hrs. 

### Determination of antibacterial activity

The disk diffusion method was performed using the standard procedure (CLSI) [19]. The three extracts in 5 dilutions (6.25, 12.5, 25, 50, 100 mg /ml) were tested by the disk diffusion method. Bacterial suspensions with a turbidity equivalent to 0.5 McFarland (1.5×10^8^) CFU/ml in BHI broth (Brain Heart infusion broth) (Merck, Germany) were prepared. Then, the suspension was cultured on Muller Hinton Agar (Merck, Germany) [18], [19], [20], [21]. The prepared disks of the different concentrations were placed on the medium. Then, disks containing DMSO were used as the negative control and disks with various antibiotics Penicillin (10 mg), Gentamicin (10 mg) or Vancomycin (30 mg) (Mast England) were used as the positive controls. The plates were incubated at 37°C for 24 hrs. Finally, the zones of inhibition were determined [18], [19], [20], [21]. 

Extracts that showed potent antibacterial activity were further tested to determine the Minimum Inhibitory Concentration (MIC) by the broth microdilution method. To determine the MIC of the extracts, a solution of 5 mg/ml triphenyltetrazolium (TPTZ) chloride was used. The test was repeated three times to obtain a mean value [22].

### Statistical analysis

The Statistical Package for the Social Sciences (SPSS) version 20, ANOVA and Duncan’s test were used to analyze the data. 

## Results

### Disk diffusion method

The alcoholic extract showed more effective antibacterial action compared to the other extracts (Table 1 (Tab. 1), Table 2 (Tab. 2)). The activity between samples of spring and summer did not show significant differences.

### Minimum inhibitory concentration

The alcoholic extract of leaves and stems was the most effective on *Streptococcus mutans* at a concentration of 0.19 ppm, and the least effective on *Staphylococcus epidermidis* at concentrations of 12.5 ppm. The hydroalcoholic extract of leaves and stems showed the highest activity on *S. aureus* and *S. mutans* at concentrations of 3.12 and 6.25 ppm, respectively, and the lowest activity on *S. epidermidis* and *N. sicca* at a concentration of 12.5 ppm (Table 3 (Tab. 3)).The aqueous extract of leaves and stems did not show antimicrobial activity.

## Discussion

Because of the increasing antibiotic resistance rates, manufacturing a new antimicrobial compound is a priority for researchers [2], [3], [4], [5], [6], [7], [8], [9], [10], [11], [12], [13], [14], [15], [16], [17], [18], [19], [20], [21], [22], [23]. Since medicinal plants have fewer side effects and are less expensive, they can be used as a source of antibiotics. The diverse geographical and climatic conditions in Iran have brought forth a rich and varied flora. Many of these plants have medicinal properties, such as antibacterial activity [24]. The use of plants of the family Thymelaeaceae has a long history of treating diseases. The genus Daphne is the most medicinally imporant taxon and the most widely used [6], [7], [8], [9], [10]. The highest antimicrobial potentials were observed for the alcoholic extract of leaves. *S. mutans* was most sensitive species, and the most resistant bacteria were *S. epidermidis* and *P. aeruginosa*. In agreement with a study by Tayoub et al. [3] our results showed biological activity of the leaves and stems. The results of the study by Javidnia et al. showed bioactive compounds in roots, stems and leaves of *Daphne* species [12], which were similar to the results of the present study. Other studies on the genus *Daphne* showed that the ethanol extract of the stems and leaves contains compounds with antibacterial properties. The study by Tayoub et al. demonstrated the antibacterial effect of an ethanol extract of the leaves and stems of *Daphne oliefolia* Lam against *Escherichia coli*, *Bacillus subtilis*, and *P. aeruginosa*, while Javadnia et al. found antibacterial and antifungal activity of an ethanolic extract of the leaves and stems of *Daphne mucronata* against four species of Gram-positive and Gram-negative bacteria [3]. The results obtained by Javidnia showed that ethanolic extracts were active against *Escherichia coli* and *S. aureus*, but ethanolic root extracts were the most effective against *S. aureus* and *Bacillus subtilis *[12]. In that study, the leaf and stem extract of *Daphne mucronata* had no effect on *P. aeruginosa* even at high concentrations; our findings were similar. Abidi et al. [23] found anti pseudomonal activity of *Daphne mucronata* 5% aqueous extracts using the disk diffusion assay. *Daphne mucronata* produced a 12 mm zone of inhibition, a biofilm reduction of 40.1%, and biofilm removal of 46% [17]. Cottiglia et al. showed the antibacterial effect of *Daphne gnidium L.* against different types of bacteria [11]. The stems of *Daphne gnidium L.* contain 4- coumarin and 7-flavonoid, which could explain the antibacterial activity [11]. A different study also showed that the leaves of Daphne ginidium contain flavonoids and phenolic compounds [17].

## Conclusion

Antimicrobial activity studies have shown that *Daphne mucronata* is very suitable for pharmacognostic and phytochemical studies. Future biochemical studies should test the effective extracted compounds from *Daphne mucronata* in various diseases.

## Notes

### Competing interests

The authors declare that they have no competing interests.

## Figures and Tables

**Table 1:**
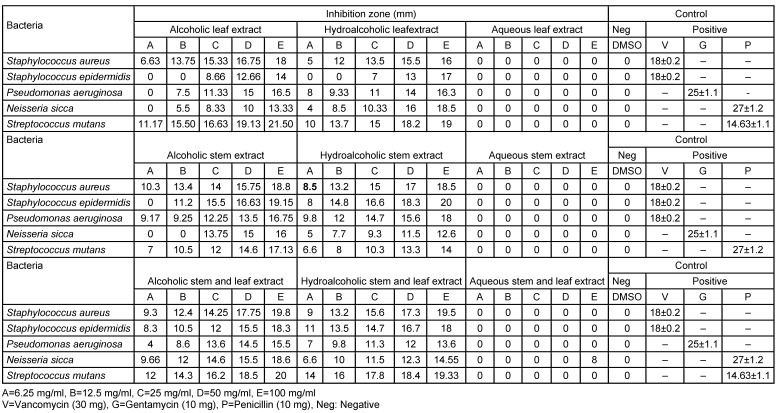
Zone of inhibition of alcoholic, hydroalcoholic and aqueous extracts of *D. mucronata* in springtime (each n=1)

**Table 2:**
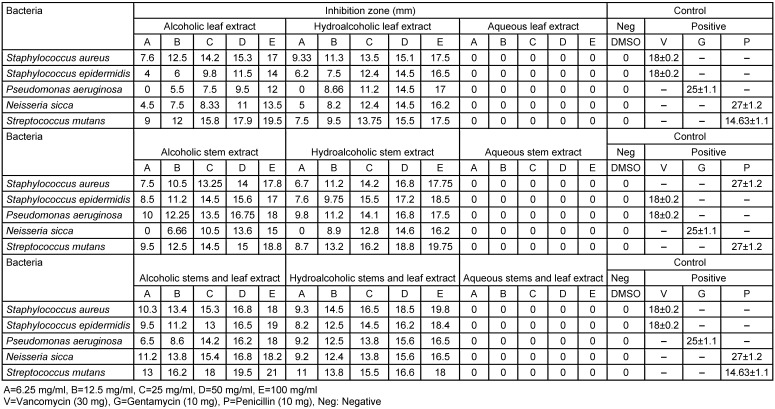
Zone of inhibition of the alcoholic, hydroalcoholic, aqueous extracts of *D. mucronata* harvested in summer

**Table 3:**
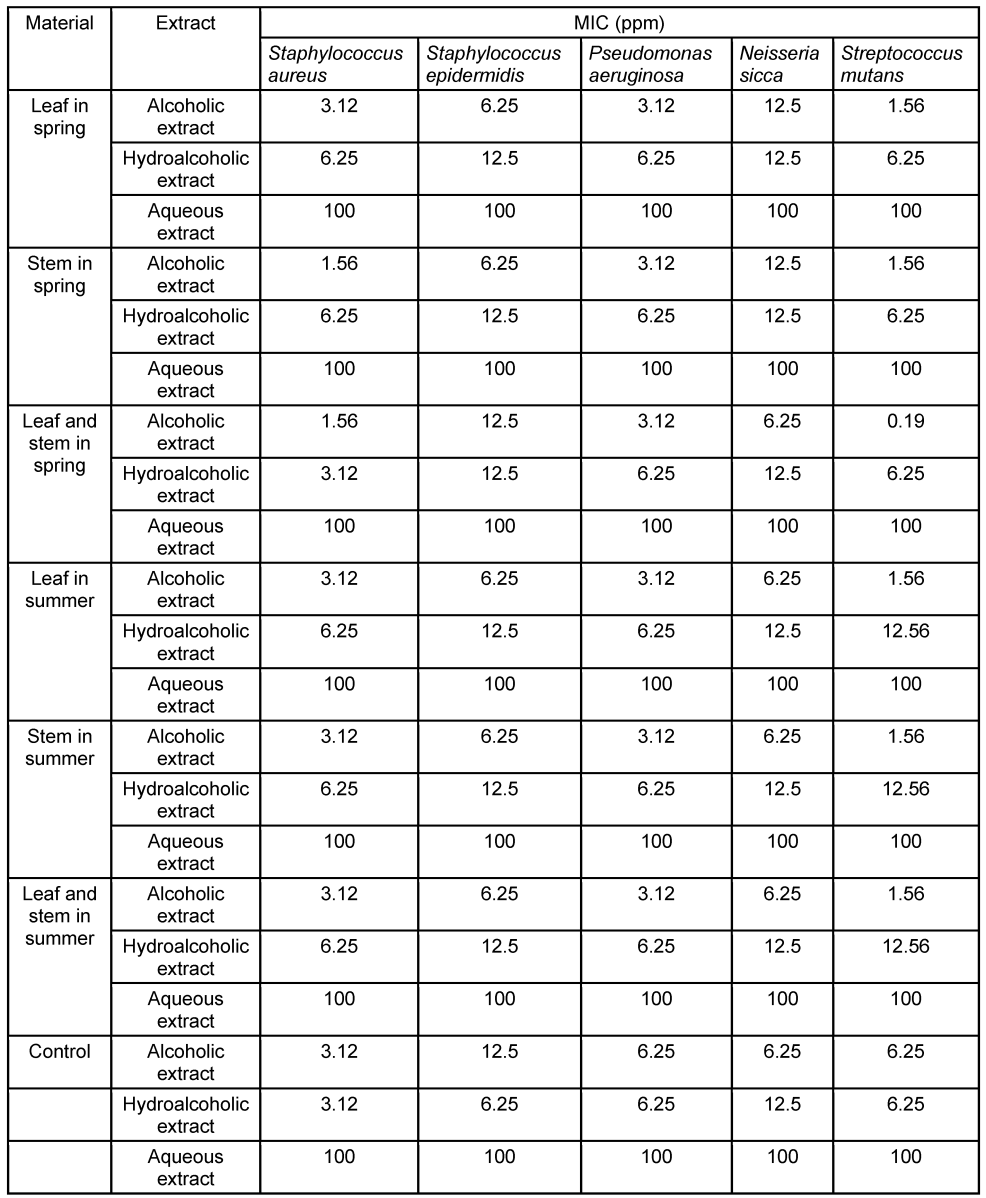
Results (mean) of minimum inhibitory concentration (MIC) in different dilutions of *Daphne mucronata* extracts

## References

[1] Houshmand B, Mortazavi H, Alikhani Y, Abdolsamadi H, Ahmadimotemayel F, Zaremahmoudabadi R (2011). In vitro evaluation of antibacterial effect of myrtus extract with different concentrations on some oral bacteria. J Mashad Dent Sch.

[2] Kasper DL, Fauci AS (2013). Harrison's Infectious Diseases.

[3] Tayoub G, Abu Alnaser A, Shamma M (2012). Microbial inhibitory of the Daphne oleifolia lam. ethanolic extract. Int J Med Arom Plants.

[4] Buhner SH (2012). Herbal Antibiotics: Natural Alternatives for Treating Drug-resistant Bacteria.

[5] Buhner SH (1999). Herbal Antibiotics: Natural Alternatives for treating Drug-Resistant Bacteria.

[6] Hedayati S, Azizi F (2005). The effect of Daphne Mucronata exteract on TNF-α and its receptor on cultured human monocytes. Cell J (Yakhteh).

[7] Kupchan SM, Baxter RL (1975). Mezerein: antileukemic principle isolated from Daphne mezereum L. Science.

[8] Liou Y, Hall I, Lee K (1982). Antitumor agents LVI: The protein synthesis inhibition by genkwadaphnin and yuanhuacine of P‐388 lymphocytic leukemia cells. J Pharm Sci.

[9] Nasipuri R, Ramstad E (1973). Isolation of Daphnetin-8-β-glucoside from Daphne Papyracea. J Pharm Sci.

[10] Mehdi H, Razieh Y, Marjan Zarif Y, Laleh Hoghooghi R, Fereidoun A (2011). A new diterpene extracted from Daphne mucronata, effects on human K562 and CCRF-CEM cell lines. J Cancer Ther.

[11] Cottiglia F, Loy G, Garau D, Floris C, Caus M, Pompei R, Bonsignore L (2001). Antimicrobial evaluation of coumarins and flavonoids from the stems of Daphne gnidium L. Phytomed.

[12] Javidnia K, Miri R, Jahromi RBNNK (2003). A preliminary study on the biological activity of Daphne mucronata Royle. DARU J Pharm Sci.

[13] Satô M, Hasegawa M (1972). Biosynthesis of dihydroxycoumarins in Daphne odora and Cichorium intybus. Phytochem.

[14] Zhao YX, Huang SZ, Ma QY, Mei WL, Dai HF (2012). Two new daucane sesquiterpenoids from Daphne aurantiaca. Molecules.

[15] Rasool MA, Imran M, Nawaz H, Malik A, Kazmi SU (2009). Phytochemical studies on Daphne mucronata. J Chem Soc Pakistan.

[16] Nikitkova AE, Haase EM, Scannapieco FA (2013). Taking the starch out of oral biofilm formation: molecular basis and functional significance of salivary α-amylase binding to oral streptococci. Appl Environm Microbiol.

[17] Abidi S, Ahmed K, Sherwani S, Kazmi S (2014). Reduction and removal of Pseudomonas aeruginosa biofilm by natural agents. Int J Chem Pharm Sci.

[18] Mahmoudi H, Arabestani MR, Molavi M, Karamolah KS, Fahim NZ (2016). The study effects antimicrobial of Foeniculum vulgare mill and Achilles mille folium plant on bacterial pathogens causing urinary tract infections and nosocomial infection. Int J Pharmacogn Phytochem Res.

[19] Clinical and Laboratory Standards Institute (2009). Performance Standards for Antimicrobial Susceptebility Testing: Nineteenth Informational Supplement M100-S19.

[20] Mahmoudi H, Arabestani MR, Mousavi SF, Alikhani MY (2017). Molecular analysis of the coagulase gene in clinical and nasal carrier isolates of methicillin-resistant Staphylococcus aureus by restriction fragment length polymorphism. J Glob Antimicrob Resist.

[21] Mahmoudi H, Arabestani MR, Mousavi SF, Ghafel S, Alikhani MY (2015). Study of polymorphism spa gene (encoding protein A) of Staphylococcus aureus in clinical isolates and nasal carriers. Tehran Univ Med J TUMS Public.

[22] Jouda MM, Elbashiti T, Masad A, Albayoumi M (2015). The antibacterial effect of some medicinal plant extracts and their synergistic effect with antibiotics. World J Pharm Sci.

[23] Abiri R, Majnooni M, Malek Khattabi P, Adibi H (2009). Study of antimicrobial effects of Trigonella Foenum hydro-alcoholic extract on different bacterial strains. Med Lab J.

[24] Alizadeh Behbahani B, Tabatabaei Yazdi F, Shahidi F, Mohebbi M (2014). Antimicrobial effect of the aqueous and ethanolic Avicennia marina extracts on Staphylococcus epidermidis, Streptococcus pyogenes and Pseudomonas aeruginosa "in vitro". Iran South Med J.

